# Deletion of *Col15a1* Modulates the Tumour Extracellular Matrix and Leads to Increased Tumour Growth in the *MMTV-PyMT* Mouse Mammary Carcinoma Model

**DOI:** 10.3390/ijms22189978

**Published:** 2021-09-15

**Authors:** Guillermo Martínez-Nieto, Ritva Heljasvaara, Anne Heikkinen, Hanne-Kaisa Kaski, Raman Devarajan, Otto Rinne, Charlotta Henriksson, Emmi Thomson, Camilla von Hertzen, Ilkka Miinalainen, Heli Ruotsalainen, Taina Pihlajaniemi, Sanna-Maria Karppinen

**Affiliations:** 1 Oulu Center for Cell-Matrix Research, Faculty of Biochemistry and Molecular Medicine, University of Oulu, 90220 Oulu, Finland; Guillermo.martineznieto@utu.fi (G.M.-N.); ritva.heljasvaara@oulu.fi (R.H.); anne.heikkinen@oulu.fi (A.H.); hanne-kaisa.kaski@fimnet.fi (H.-K.K.); raman.devarajan@oulu.fi (R.D.); Otto.Rinne@mehilainen.fi (O.R.); charlotta.henriksson@oulu.fi (C.H.); emmi.thomson@oulu.fi (E.T.); camilla.vonhertzen@oulu.fi (C.v.H.); heli.ruotsalainen@oulu.fi (H.R.); taina.pihlajaniemi@oulu.fi (T.P.); 2 Biocenter Oulu, University of Oulu, 90220 Oulu, Finland; ilkka.miinalainen@oulu.fi

**Keywords:** angiogenesis, breast cancer, collagen XV, ECM remodelling, fibrosarcoma, MMTV-PyMT, restin, syngeneic tumour models, tumour stroma

## Abstract

Basement membrane (BM) zone-associated collagen XV (ColXV) has been shown to suppress the malignancy of tumour cells, and its restin domain can inhibit angiogenesis. In human breast cancer, as well as in many other human carcinomas, ColXV is lost from the epithelial BM zone prior to tumour invasion. Here, we addressed the roles of ColXV in breast carcinogenesis using the transgenic *MMTV-PyMT* mouse mammary carcinoma model. We show here for the first time that the inactivation of *Col15a1* in mice leads to changes in the fibrillar tumour matrix and to increased mammary tumour growth. ColXV is expressed by myoepithelial and endothelial cells in mammary tumours and is lost from the ductal BM along with the loss of the myoepithelial layer during cancer progression while persisting in blood vessels and capillaries, even in invasive tumours. However, despite the absence of anti-angiogenic restin domain, neovascularisation was reduced rather than increased in the ColXV-deficient mammary tumours compared to controls. We also show that, in robust tumour cell transplantation models or in a chemical-induced fibrosarcoma model, the inactivation of *Col15a1* does not affect tumour growth or angiogenesis. In conclusion, our results support the proposed tumour suppressor function of ColXV in mammary carcinogenesis and reveal diverse roles of this collagen in different cancer types.

## 1. Introduction

The extracellular matrix (ECM), together with blood vessels, immune cells, fibroblasts and signalling molecules, forms the tumour microenvironment, which significantly contributes to cancer development and progression. Compared to the ECM of healthy tissues, the architecture of tumour ECM is commonly disorganized, and its remodelling is dysregulated, which can drive tumour progression by activating cell proliferation and invasion [1,2]. 

Collagen (Col) XV is a non-fibrillar basement membrane (BM) zone-associated proteoglycan which, together with another BM proteoglycan, ColXVIII, forms a subgroup of multiplexins (multiple triple-helix domains with interruptions) within the collagen superfamily [3,4]. These two collagens share a similar multidomain structure, for example, both contain an anti-angiogenic C-terminal domain termed restin in ColXV and endostatin in ColXVIII. However, the two multiplexins have many differences in terms of structure, tissue distribution, localisation and physiological functions, as summarised in recent review articles [5,6,7,8]. ColXVIII is an integral component of the BM, whereas ColXV localises at the interface between the BM and fibrillar collagen network and has an important role in maintaining the integrity of the ECM [5,6,7,8,9,10]. ColXV is primarily produced by cells originating from the mesenchyme, including fibroblasts, myocytes, adipocytes and nerve cells, and is strongly expressed, for example, in the heart and skeletal muscles [5]. It is also abundantly expressed by endothelial cells and deposited to vascular BM zones, securing the integrity of the perivascular ECM and capillaries [5,11]. Genetically modified model organisms highlight the functional importance of ColXV in the muscular, cardiovascular and neuromuscular systems [5,10,11]. 

The anti-angiogenic and anti-tumourigenic functions of ColXVIII-derived endostatin have been extensively studied in different cancers [7,8,12], whereas substantially less is known about the roles of ColXV and its restin domain in cancer. The expression and localisation of ColXV is altered in human breast, colon and skin carcinomas: it disappears from the epithelial BM zones in invasive tumours and, in some cases, becomes expressed by cancer-associated fibroblasts and inflammatory cells in the desmoplastic tumour stroma [13,14,15,16]. Instead, ColXV persists or is even upregulated in the vascular BMs of tumours [13,14,16,17]. Based on the localisation of ColXV in the malignant fibrillar stroma and in tumour vasculature, and its known function in supporting ECM in healthy tissues [5,6,10,11], it has been postulated to stabilise tumour vessels and promote the organisation of a fibrotic tumour stroma. In respect to tumour angiogenesis, it has been shown that the recombinant restin domain of human ColXV impedes neovascularisation in vivo in renal carcinoma xenografts [18]. Mechanistically, restin inhibits the proliferation and migration of endothelial cells, and induces their apoptosis in vitro [18,19,20]. 

An interesting hypothesis on the role of ColXV as a tumour suppressor was launched in 2003 [21]. This hypothesis is based on observations of the presence of the ColXV gene in a tumour suppressor locus in the mouse and human genome, and on cell studies showing that the deletion of a chromosome 4 locus containing *Col15a1* in mouse fibroblasts results in a malignancy of tumour cell hybrids and compromises the formation of collagenous tumour stroma [22,23]. These observations were followed up by studies showing that the overexpression of full-length ColXV cDNA in human cervical cancer cells (which normally do not express ColXV) significantly inhibits tumour cell growth in soft agar and in vivo without affecting tumour angiogenesis in xenografts; furthermore, the overexpression of the restin domain alone in tumour cells does not inhibit the growth of subcutaneous tumours [24,25].

The functions and significance of ColXV in different cancers are incompletely understood. The previously proposed tumour suppressor [6,21,24,25,26] and anti-angiogenic roles [18,19,20,25] of ColXV and restin are largely based on in vitro studies. Here, we wanted to further clarify these suggestions by using in vivo mouse tumour models. Hence, we crossed *Col15a1* knockout mice with a transgenic mouse model with mammary tumour virus promoter-driven overexpression of the polyoma middle T antigen (*MMTV-PyMT*) in mammary epithelial cells. In addition, to obtain a broader view on ColXV’s roles in tumourigenesis, we monitored its impact in other selected experimental mouse tumour models, namely syngeneic lung cancer, melanoma and fibrosarcoma transplants, as well as chemical-induced subcutaneous fibrosarcoma. We show that the deletion of the gene encoding ColXV leads to increased tumour growth in the *MMTV-PyMT* model but does not affect tumour growth in the other models. Importantly, we observed that ColXV is needed for the proper organisation of the tumour matrix. Moreover, the ablation of ColXV, including its anti-angiogenic restin domain, does not allow for more potent tumour angiogenesis in the used experimental models but affects capillaries in mammary tumours. In conclusion, our study highlights the role of ColXV as an ECM organizer in the breast tumour microenvironment and reveals potentially diverse functions in different cancer types.

## 2. Results

### 2.1. ColXV in Mouse Mammary Carcinogenesis

**Figure 1:**
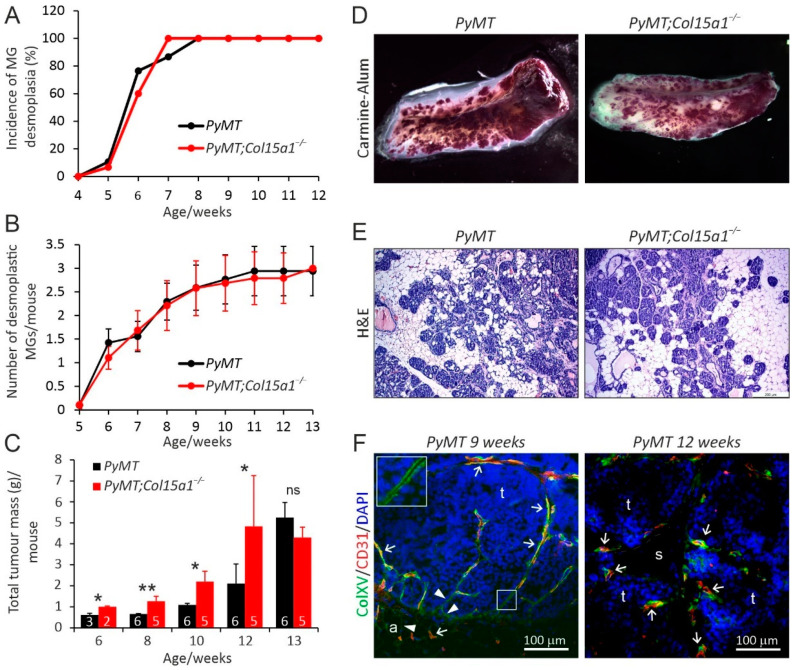
Mammary carcinogenesis in the wild-type *MMTV-PyMT* (*PyMT*) and ColXV-deficient *PyMT;Col15a1^−/−^* mice. The incidence (**A**) and number (**B**) of desmoplastic mammary glands (MG) per mouse at the age of 4–13 weeks. Abdominal and thoracic MGs were palpated weekly. (**C**) Total tumour burden in the MGs of *PyMT* and *PyMT;Col15a1^−/−^* mice at indicated ages, measured as total weight of the dissected four abdominal and two thoracic MGs. The numbers of mice included in the analyses are shown in the bars. (**D**) Representative images of Carmine Alum-stained whole mount preparations of MGs at week 11, and (**E**) haematoxylin-eosin (H&E)-stained MG sections at week 6. (**F**) Representative images of ColXV and CD-31 double immunofluorescence staining in mammary tumours of control *PyMT* mice at the age of 9 and 12 weeks. ColXV localises in the BMs of tumour nests (arrowheads and magnification in insert) and in vascular BMs, where it often overlaps with the CD-31 signal (arrows). a, adipocyte; s, tumour stroma; t, tumour tissue. Fluorescent signals: ColXV, green; CD-31, red; nuclei, DAPI, blue. The scale bar in E is 200 µm and in F 100 µm. The error bars in B and C indicate s.e.m. * *p* < 0.05; ** *p* < 0.01; ns, not significant.

To investigate the in vivo roles of ColXV in mammary tumourigenesis, we crossed *Col15a1^−/−^* mice with the *MMTV-PyMT* model and compared tumour development in the *PyMT;Col15a1^−/−^* crosses with the wild-type *MMTV-PyMT* (*PyMT*) mice from 4 weeks up to 14 weeks of age. We did not observe differences in the incidence or number of palpable, desmoplastic mammary glands between the two mouse lines during the examination period (Figure 1A,B). Although considerable variation was observed in the weights of individual tumourous glands in both genotypes, the knockout mice showed a significantly increased total tumour burden at weeks 6–12 compared with the *PyMT* controls; however, this difference levelled off in 13-week-old mice (Figure 1C). A visual inspection of whole mount Carmine Alum or haematoxylin-eosin-stained glands did not reveal obvious differences in the amount of tumourous tissue between the *PyMT* and *PyMT;Col15a1^−/−^* mice at 6, 9, 11 or 13 weeks of age (Figure 1D,E, some data not shown), most likely because of the substantial variation in the amount of tumourous tissue in different glands in both genotypes. Quantification of the proliferation marker protein Ki67 and the proliferating cell nuclear antigen (PCNA) signals in tumours showed considerable variation between, and even within, the specimens; however, both antibodies revealed a trend of more active tumour cell proliferation in *PyMT;Col15a1^−/−^* samples than in *PyMT* samples at weeks 6–9 (Supplementary Figure S1). Conversely, at later stages of tumourigenesis, at weeks 10–14, cancer cells in control *PyMT* tumours divided more than those in the knockout samples (Supplementary Figure S1). In summary, we observed an increased mammary tumour burden in the ColXV-deficient mice; however, our analyses of tumour cell proliferation did not unambiguously explain this change.

Immunofluorescence staining of wild-type *PyMT* mammary tumours (Figure 1F, Supplementary Figure S2) showed that the ColXV signal surrounds normal mammary ducts, where it resides juxtaposed and sometimes overlaps with the myoepithelial cell marker alpha smooth muscle actin (αSMA) (Supplementary Figure S2A–C). A thin ColXV signal can also be detected around mammary gland adipocytes (Figure 1F, Supplementary Figure S2A–C). In mammary ducts that are partially or completely filled with tumour cells, the ColXV signal still encircles the tumour nests (Figure 1F), whereas the αSMA signal has disappeared from these sites, as indicated during mammary tumour progression (Supplementary Figure S2D–F) [27]. In these samples, the ColXV signal is strong in the BMs of capillaries and larger blood vessels (Figure 1F), with the latter also showing αSMA-positive smooth muscle layers (Supplementary Figure S2D–F). In invasive mammary tumours (Figure 1F, Supplementary Figure S2G–I), the ColXV signal associates with blood vessels and capillaries, where it localises in close proximity or overlaps with the endothelial marker CD-31. ColXV is mainly lost from the borders of invasive tumours (Figure 1F, Supplementary Figure S2G–I). Occasionally, faint and discontinuous ColXV signals can be seen at the edges of advanced tumours, sometimes colocalising with faint αSMA signals in these sites (Supplementary Figure S2J,K). A strong αSMA signal was detected also in elongated structures in the interstitial tumour stroma, presumably representing cancer-associated fibroblasts; however, a ColXV signal was not observed in these locations (Supplementary Figure S2J). In addition to BM-associated staining, ColXV sometimes showed a cytoplasmic type of staining in the myoepithelial layer surrounding the tumour islands (Supplementary Figure S2L), indicating that these cells actively produce ColXV and deposit it into the BM. The ColXV antibody did not reveal signals in *PyMT;Col15a1^−/−^* samples, and the secondary antibodies did not show unspecific binding in *PyMT* samples, confirming the specificity of the antibodies used in the immunofluorescence staining (Supplementary Figure S2M–O). In summary, in PyMT oncogene-driven mammary tumours, ColXV is expressed by myoepithelial cells and localises around tumour nests in early-stage tumours and in ductal BMs in normal mammary ducts adjacent to tumour tissue. ColXV is also produced by endothelial cells and deposited to tumour vascular BMs. When tumours progress in malignancy, ColXV disappears from the ductal BM but prevails in the tumour vasculature. These first observations made on ColXV expression in mouse mammary tumours are in accordance with ColXV localisation in ductal carcinomas of the human breast, except for the deposits of ColXV signal in the interstitial stroma of human tumours [15].

**Figure 2:**
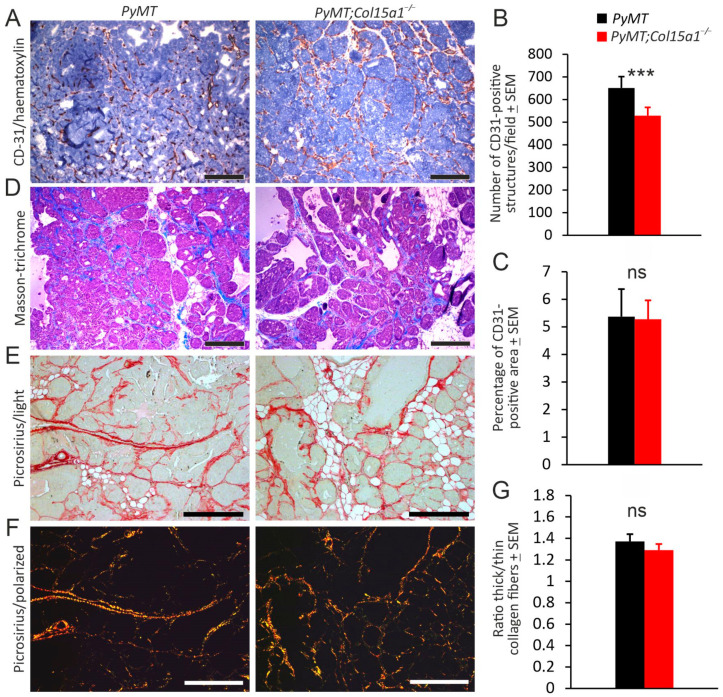
Angiogenesis and fibrosis in *MMTV-PyMT* mammary tumours. (**A**) Immunohistochemistry for CD-31 (red) in tumours of 12-week-old control *PyMT* and *PyMT;Col15a1^−/−^* mice. (**B**) Manual counting of CD-31-positive structures in tumours. For both genotypes (*n* = 9), four random microscopic fields were analysed at 100× magnification. (**C**) ImageJ analysis of CD-31-positive areas in the same microscopic fields. Quantification of the signal in the light microscopy images of tumour samples stained with Masson’s trichrome (**D**) and picrosirius red (**E**) revealed comparable fibrillar collagen content in the control and *PyMT;Col15a1^−/−^* tumours. (**F**,**G**) Quantification of the birefringence of picrosirius red signals captured under polarised light showed a slightly reduced ratio of thick (red) to thin (green) collagen fibres in the *PyMT;Col15a1^−/−^* tumours in comparison to the *PyMT* control tumours. Samples were collected from mice at 10–14 weeks of age (*n* = 8 mice for both genotypes; one tumour sample per mouse was analysed). Four microscopic fields per tumour at 100× magnification were analysed. Scale bars in (**A**,**D**–**F**), 200 µm. Error bars in (**B**,**C**,**G**) indicate s.e.m. *** *p* < 0.001; ns, not significant.

We next focused on angiogenesis and ECM remodelling in *PyMT* mammary tumours in the absence of ColXV. Counting of intratumoural blood vessels in highly vascularised areas of mammary tumours showed significantly (approximately 20%) less CD-31-positive structures in the *PyMT;Col15a1^−/−^* tumours than in the *PyMT* control tumours on average, potentially indicative of impaired tumour angiogenesis in the absence of the perivascular vessel stabiliser ColXV [10,11]. However, quantification of the CD-31 signals in the same microscopic fields by automatic image analysis showed equally large CD-31-positive areas in the samples, suggesting that *PyMT;Col15a1^−/−^* tumours may contain fewer but larger blood vessels than control tumours (Figure 2A–C). 

The content of fibrillar collagen in the tumour tissues was analysed using Masson’s trichrome and picrosirius staining and imaged by light microscopy. Neither method showed obvious differences in the structure or amount of fibrillar stroma between the genotypes (Figure 2D,E). Nevertheless, the examination of picrosirius red-stained sections under polarised light, followed by quantification of birefringence, revealed that the ratio of thick to thin collagen fibres was reduced to some extent, but not significantly, due to *Col15a1* inactivation (Figure 2F,G).

**Figure 3:**
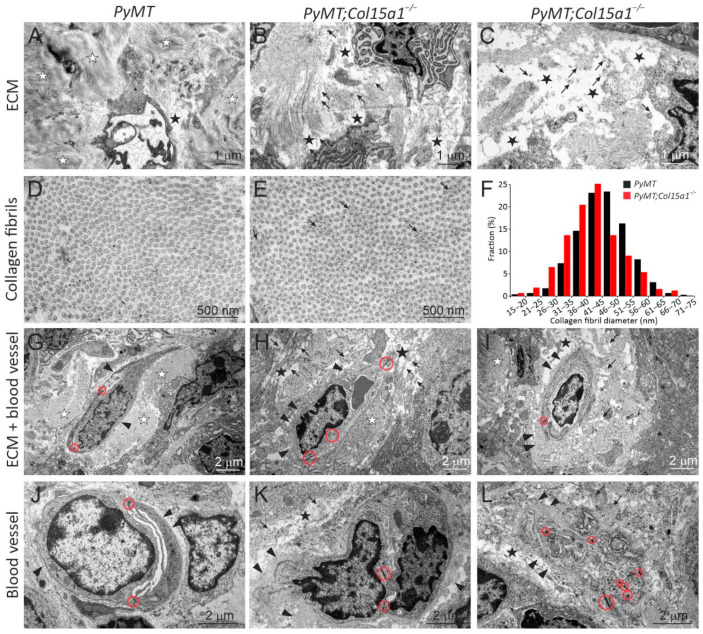
Ultrastructural analysis of mammary tumour stroma. Transmission electron microscopy shows that, in the *PyMT* control tumours (**A**,**G**), the stromal ECM is filled with clearly outlined collagen fibres (white stars), whereas, in the *PyMT;Col15a1^−/−^* tumours (**B**,**C**,**H**,**I**), the ECM appear sparse (black stars), with accumulations of non-fibrillar protein aggregates (arrows). These protein aggregates accumulated also within collagen fibres in the *PyMT;Col15a1^−/−^* tumours (**E**). The collagen fibril diameter in *PyMT;Col15a1^−/−^* tumours is slightly decreased and variable (**E**,**F**) when compared to *PyMT* control tumours (**D**,**F**). For calculating fibril diameter fractions, 893–1032 fibrils/genotype and 121–265 fibrils/tumour sample were measured. The capillary BM (arrowheads) is well defined in the *PyMT* control tumours (**G**,**J**) but thickened with ECM protein deposits in the *PyMT;Col15a1^−/−^* tumours (**H**,**I**,**K**,**L**). No obvious difference was found in the endothelial cell–cell junctions (open red circles) in the tumour capillaries between genotypes (**G**–**L**). Three mammary tumours from two mice of both genotypes (*n* = 6 + 6), harvested at the age of 10 weeks, were analysed by electron microscopy.

We then analysed the tumour samples at the ultrastructural level using transmission electron microscopy. Stromal ECM in the *PyMT* control tumours contained large collagen bundles and smaller-order fibrillar and mesh-like protein structures that typically filled the extracellular space (Figure 3A,D,G). In the *PyMT;Col15a1^−/−^* tumours, the stromal ECM was more often loosely packed than in the controls, and contained non-fibrillar protein deposits which also accumulated on collagen fibrils (Figure 3B,C,E,H,I). The collagen content appeared slightly decreased in the *PyMT;Col15a1^−/−^* samples, however, the collagen fibril density in the bundles was similar in both genotypes (Figure 3D,E), being 209.5 ± 43.2 fibrils/mm^2^ in *PyMT* and 214.1 ± 47.9 fibrils/mm^2^ in the *PyMT;Col15a1^−/−^* samples; *p* = 0.86. In addition, in the *PyMT;Col15a1^−/−^* tumour stroma, the collagen fibrils were slightly, although not statistically significantly, smaller in diameter and were also more variable in size than fibrils in the control samples (Figure 3D–F; for *PyMT*, M (mean fibril diameter) is 46.1 ± 7.9 nm and SD% (standard deviation of fibril diameter in %) is 17.2 ± 1.8%; for *PyMT;Col15a1^−/−^* M is 42.2 ± 8.4 nm and SD% is 19.9 ± 1.2%); *t*-test for M, *p* = 0.09 and for (SD%) *p* = 0.012). These analyses supported the observations made by picrosirius staining on the presence of immature fibres in the ColXV-deficient tumours (Figure 2G). The most consistent, exclusive and highly prevalent finding in the *PyMT;Col15a1^−/−^* tumours was accumulation of non-fibrillar protein aggregates on the capillary BM, making it locally excessively thick (Figure 3H,I,K,L), similar to those observed in the heart tissue capillaries of the *Col15a1^−/−^* mice [10]. *PyMT;Col15a1^−/−^* tumour capillaries were typically covered with regions of both protein aggregates and normal looking BM. Aggregates were not evenly distributed over the entire length of the *PyMT;Col15a1^−/−^* tumour vessels, and their thickness varied from 100 nm to even 1500 nm (mean thickness 297 ± 220nm). Notably, these capillary BM aggregates were present in all analysed *PyMT;Col15a1^−/−^* tumours but were not observed in the control tumours (Fig. 3G-L). Morphological changes in the ColXV-deficient capillaries have been previously reported in cardiac and skeletal muscle [10,11,28]. In the tumour stroma, however, the integrity of endothelial cells was compromised in both genotypes, and reliable assessment of recurrent changes, such as those in endothelial cell–cell junctions, was not possible in the *PyMT;Col15a1^−/−^* tumours in relation to the *PyMT* control tumours (Figure 3G–L). In summary, our investigations on tumour ECM remodelling in *PyMT* tumours lacking ColXV expression demonstrated that this collagen has a role as an important organiser of fibrillar tumour stroma and perivascular ECM. In addition, the data from this cancer model confirm the previous observations that the ablation of ColXV/restin does not lead to enhanced tumour neovascularisation.

### 2.2. Other In Vivo Tumour Models

**Figure 4:**
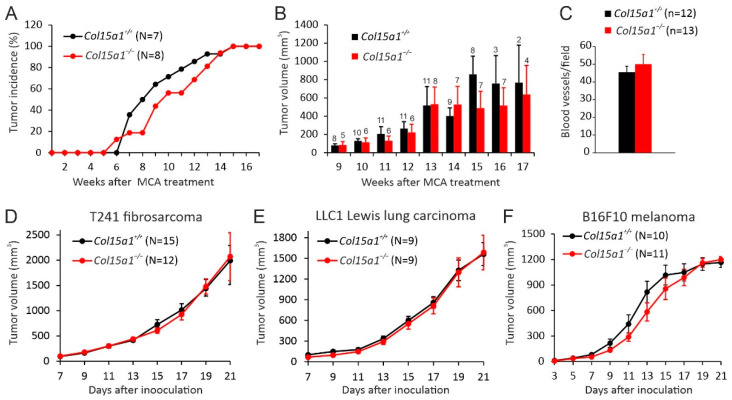
MCA-induced fibrosarcoma model and syngeneic transplantation models. (**A**) Incidence of MCA-induced subcutaneous fibrosarcomas in *Col15a1^+/+^* control and *Col15a1^−/−^* knockout mice, presented as the percentage of mice bearing at least one persistent tumour of 3 mm in diameter. (**B**) Tumour volumes at different time points. Numbers of tumours included in the analysis at different time points are shown above the bars. (**C**) Microvessel densities in MCA-induced fibrosarcomas were determined in 10 random fields of CD-31-stained fibrosarcomas (*Col15a1^+/+^ n* = 12, *Col15a1^−/−^ n* = 13) collected 13–17 weeks after MCA treatment at 200× magnification. (**D**–**F**) Growth curves of subcutaneous syngeneic tumour transplants in the *Col15a1^+/+^* control and *Col15a1^−/−^* mice. Numbers of mice (N) and tumours (n) included in the experiments are indicated in panels. In (**B**–**F**), error bars indicate s.e.m.

The functions of ColXV in carcinogenesis were assessed using other types of experimental mouse tumour models in addition to the *MMTV-PyMT* model. Thus, subcutaneous fibrosarcomas were induced in the *Col15a1^+/+^* control and *Col15a1^−/−^* knockout mice, both in the C57BL/6JOlaHsd background, with a 3-methylchlolantrene (MCA) treatment. This model was chosen because it represents a cancer of a mesenchymal cell origin, a cell type associated with prominent ColXV expression [5], and follows the natural stepwise development of cancer with chronic inflammation and accumulating mutations [29,30]. Tumours appeared in the flanks of the mice after a similar latency period of 5–6 weeks in both genotypes and, even if linear modelling showed that the *Col15a1^−/−^* mice have a delay in the tumour incidence (i.e., in the percentage of mice bearing at least one persistent tumour of 3 mm in diameter), this difference between the genotypes was not significant, and all mice developed a measurable tumour at least in one flank by week 15 from the carcinogen treatment (Figure 4A). In addition, tumour size measurements showed a trend of reduced growth in the *Col15a1^−/−^* mice at the late monitoring phase; however, the differences between the genotypes were not significant at these or any other time points (Figure 4B). The effect of ColXV deficiency on tumour angiogenesis was evaluated by calculating the CD-31-positive intratumoural vessels in the control and knockout tumours. This analysis showed that ColXV deficiency did not alter the average number of microvessels in the MCA-induced fibrosarcomas (Figure 4C). 

Moreover, we assessed the influence of *Col15a1* deletion in tumour growth using common syngeneic mouse models for the C57BL6 background. None of the tested cancer cell lines, namely T241 sarcoma, LLC1 Lewis lung carcinoma or B16F10 melanoma, showed differences in primary tumour growth rates between *Col15a1^+/+^* and *Col15a1^−/−^* mice when injected subcutaneously (Figure 4D–F). Taken together, unlike the genetic *MMTV-PyMT* model, the transplantable or chemical models did not reveal significant changes in primary tumour growth or tumour angiogenesis in the absence of ColXV. 

### 2.3. COL15A1 Expression-Based Survival Analysis for Breast Cancer Patients

**Figure 5:**
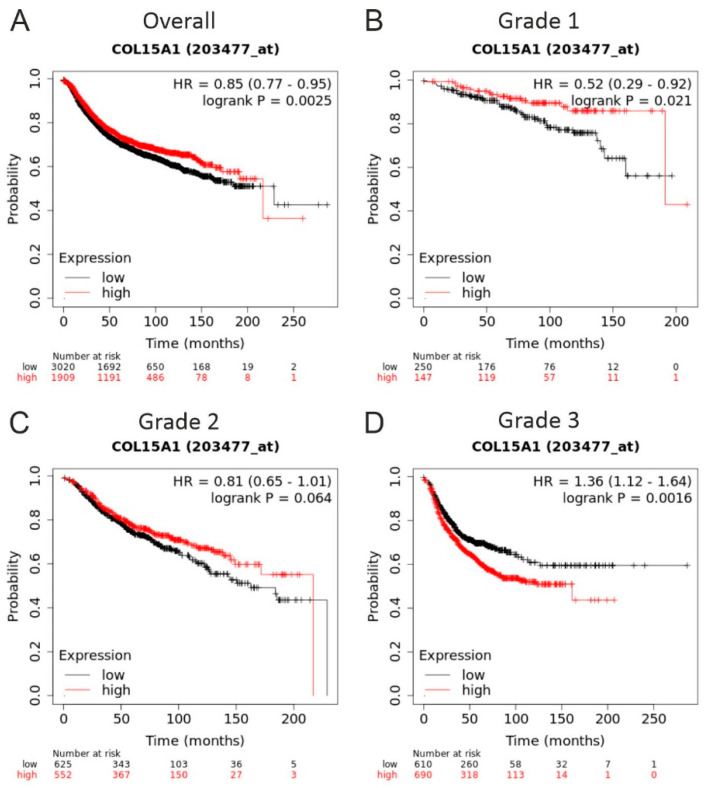
High ColXV mRNA expression is associated with good prognosis. Kaplan–Meir plots showing relapse-free survival of patients with breast cancer stratified by ColXV expression levels (probe: 203477_at). (**A**) All available breast cancer cases with ColXV expression data (*n* = 4929), (**B**) available grade 1 cases with ColXV expression data (*n* = 329), (**C**) available grade 2 cases with ColXV expression data (*n* = 1177) and (**D**) available grade 3 cases with ColXV expression data (*n* = 1300). For meta-analyses, the open access gene expression data and patients’ survival information from TCGA, GEO and EGA, compiled in a single database at ww.kmplot.com [31], were used. High ColXV, red line; low ColXV, black line. Hazard ratio (HR) and log-rank *p* values were automatically computed using the best performing threshold as the cut off. The number of patients for each analysis at different time points is also indicated in the survival graphs.

Finally, we analysed the correlation between ColXV expression levels in breast cancer tumours and patient survival in open-access databases that include genome-wide expression data and survival data of almost 8000 patients with breast cancer using the Kaplan–Meier plotter tool [31]. This analysis showed that high ColXV mRNA levels are associated significantly with a better relapse-free survival rate in low grade 1 tumours, but with poor survival in aggressive grade 3 tumours (Figure 5).

## 3. Discussion

We present here data on the impact of the BM-zone associated ColXV on carcinogenesis using our *Col15a1* knockout mice subjected to both autochthonous and transplantable tumour models. We show that the deletion of *Col15a1* affects tumour burden in the genetic *MMTV-PyMT* model; whereas, in the other studied models, the chemical subcutaneous fibrosarcoma model and syngeneic transplantation models, deletion of ColXV did not show any significant effect on tumour growth. Our key findings in the *PyMT* model are that ColXV can suppress primary tumour growth in wild-type *PyMT* mice and that the deletion of *Col15a1* leads to the aberrant organisation of the interstitial tumour matrix. These findings support the proposed tumour-suppressive and matrix-organiser roles of ColXV [6,10,21] and the subsequent data from the overexpression models [24,25,26]. 

The remodelling of tumour ECM, together with the activities of different stromal cells embedded in the ECM, significantly contributes to the tumourigenic process [2]. We previously detected a poorly organised fibrillar collagen matrix and interstitial non-fibrillar protein deposits in aged *Col15a1^−/−^* mouse hearts [10]. Here, we report a novel observation that, besides acting as a matrix organiser in non-tumourigenic tissues, ColXV has a similar role in solid tumours. We found less and loosely organised fibrillar collagen matrix in the *PyMT;Col15a1^−/−^* tumours in comparison with *PyMT* control tumours and the presence of non-fibrillar protein aggregates in the tumour stroma (Figure 3). Moreover, picrosirius red staining revealed a slight but not significant increase in the relative ratio of thin to thick collagen fibres in *PyMT;Col15a1^−/−^* tumours, indicating more immature collagen fibres in these than in the wild-type tumours (Figure 2G). In this context, we were interested in comparing our new findings with early in vitro data by Henry Harris in 1985 showing that the malignancy of fibroblast-tumour cell hybrids is suppressed due to the deposition of abundant collagenous ECM by these cells [23]. The formation of this fibrillar matrix was dependent on the chromosomal region 4A4-C3 in mouse fibroblasts [22], the region that also includes the *Col15a1* gene [32]. When this region was deleted in the cell hybrids, they regained the malignant phenotype and collagen production was reduced [23]. In summary, our current data support the previous cell hybrid studies pointing to a role for ColXV in regulating matrix remodelling in tumours and widen the view of its significance as an important matrix organiser from organ development and pathogenesis to tumourigenic processes. 

Tumour invasion is an intricate process that includes interactions between different cells, such as myoepithelial cells and immune cells, and a release of various invasive and angiogenic factors, such as matrix metalloproteases, to support tumour cell invasion and proliferation [33]. The loss of the myoepithelial cell layer and the ductal BM precede breast cancer invasion and contribute to the development of a tumour-supportive microenvironment [27,33,34]. We show here that in healthy mouse mammary tissue, ColXV is expressed by myoepithelial cells lining the mammary ducts and forms thin but continuous deposits in ductal BMs; however, in the course of mammary carcinoma progression, ColXV is lost from the ductal BM but prevails in the BMs of tumour vessels (Supplementary Figure S2). Parallel observations have been made in normal human breast tissue and in the ductal adenocarcinoma of the human breast [14] and in other carcinomas [13,15,16]. As the gradual disappearance of the myoepithelial cells is a prerequisite of breast cancer progression, the loss of ColXV in the ductal BM is probably a part of this process. Amenta et al. noticed that ColXV disappears from the ductal BM of the breast earlier than some other BM markers [14]. This may reflect the fact that, unlike the integral BM components, such as laminin, ColIV and ColXVIII, ColXV locates in the outermost layer of the BM and may thus also be easily lost from the BM structures [9,10,35]. Given that ColXV is implicated in maintaining an intact BM, its lack probably results in a fragile or fenestrated BM structure in the invasive front and in the vasculature, which promotes tumour cell invasion and metastasis [6]. Our finding on the absence of ColXV in the fibrillar, αSMA-positive stroma in the mouse mammary tumours differs from the previous observations on prominent ColXV staining in the interstitium of invasive human breast, skin and colorectal tumours [13,14,15,16].

Myoepithelial cells and the BM deposited by them separate the breast epithelium from the surrounding stroma. Moreover, they mediate communication between luminal epithelial cells and the stroma and secure the polarity of luminal epithelial cells and prevent their dissemination to the surrounding tissue [34,36]. Interestingly, luminal epithelial cells are unable to polarise when cultured alone in a ColI matrix, but undergo polarisation when cultured in the BM extract Matrigel or together with myoepithelial cells in the ColI matrix [37]. The critical BM component for cell polarisation produced by myoepithelial cells was found to be the laminin subunit a1 [37], which is known to interact with ColXV in vitro [38]. Based on these and our new findings, we speculate that myoepithelial cell-derived ColXV could be involved in the regulation of mammary epithelial cell polarity by integrating signals from the collagenous matrix through laminin to luminal epithelial cells. Thus, in the presence of ColXV, the proper architecture of the collagenous ECM is preserved and the communication between the ECM compartment and the mammary epithelium is appropriately regulated, supporting E-cadherin stabilisation and epithelial polarisation, as reported in a ColXV overexpression model of pancreatic adenocarcinoma [26]. Instead, upon depletion of myoepithelial cells and ColXV expression during cancer progression, the important regulatory link between ColI and luminal epithelium is lost, leading to epithelial-to-mesenchymal transition EMT, the upregulation of N-cadherin through the activation of the DDR1-Pyk2 pathway and tumour cell invasion, as has been shown to occur in pancreatic cancer [26]. Detailed molecular studies are needed to confirm whether ColXV is involved in the signalling routes regulating the polarity of mammary epithelial cells and epithelial-to-mesenchymal transition.

We found increased mammary tumour growth in the *PyMT;Col15a1^−/−^* mice in comparison with control *PyMT* mice almost through the follow-up period, except for in the latest examined time points, when the tumour burden was the same in both experimental groups (Figure 1C). Tumour cell proliferation analyses with two different antibodies showed a trend towards higher proliferation in early-stage *PyMT;Col15a1^−/−^* tumours collected from mice at the ages of 6–9 weeks than in controls of the same stage, but lower proliferation in late-stage tumours at weeks 10–14. ColXV overexpression has been shown to inhibit cervical carcinoma cell growth in soft agar and subcutaneous xenografts, but does not affect cell doubling time in 2D cultures [24]. Together, the findings from our knockout and previous overexpression models may imply that the tumour suppressive action of ColXV is dependent on the surrounding 3D environment and is relevant in early-stage tumours when the myoepithelial cells producing this collagen (Supplementary Figure S2) are still present in the preinvasive carcinomas. In late-stage mammary carcinomas, the lack of ColXV seems to lead to the inhibition of tumour cell proliferation and, together with compromised tumour vascularisation (Figure 2, Supplementary Figure S1), may slow down tumour growth (Figure 1C). However, this interpretation may be appropriate only for epithelial cancers; as in the chemical fibrosarcoma model, the minor differences in tumour incidence and growth between the *Col15a1^−/−^* and the wild-type control mice did not support the potential tumour suppressive functions of ColXV. Moreover, none of the transplantation carcinoma experiments conducted here, not even the epithelial LLC1 lung carcinoma, revealed tumour suppressive actions for ColXV, which is likely at least partly due to the very fast growth rates of subcutaneous tumours in these models. In summary, ColXV appears to play complex, stage-dependent roles in the regulation of carcinogenesis, and more detailed analyses in sophisticated experimental models are needed to reveal its molecular mechanisms in epithelial cancers.

Our data of increased mammary tumour growth in the *PyMT;Col15a1^−/−^* mice and the previous data on diminished ColXV expression in human breast cancer [14] raise the question of the relation between ColXV levels and patient survival. Kaplan–Meier survival analysis [31] showed that higher expression levels of *Col15a1* gene transcripts in low grade tumours associate with better relapse free survival, while in high grade tumours they associate with poor survival (Figure 5). In light of the previous data [6,14,24] and our results of the *PyMT* model, we propose that, in healthy mammary duct epithelium and low-grade tumours, ColXV is actively produced by myoepithelial cells and deposited into ductal BM, serving as a tumour suppressor and preventing the transformation of epithelial cells and their subsequent invasion. However, in high-grade tumours, myoepithelial cells are lost, and ColXV is now derived from stromal cells, including the endothelial cells of highly vascularised tumours and other cell types in the interstitium between the tumour nests, as has been previously reported in human breast and other carcinomas [13,14,16].

The neovascularisation of tumours is a prerequisite for their growth and metastasis [39]. We have shown that the deletion of *Col15a1* leads to abnormal and leaky capillaries in cardiac and skeletal muscles [10,11,28]. The tumour vasculature differs from the normal vasculature by acquiring an irregular shape and size and by being fragile and leaky, hence facilitating the intravasation of disseminating tumour cells [39,40]. Compared with intact healthy vessels, we found morphological abnormalities in the capillaries of both control *PyMT* and *PyMT;Col15a1^−/−^* tumour samples (Figure 3). Therefore, we could not make firm conclusions on the potential effects of ColXV deletion on capillary structure and function. However, based on the analysis of microvessel density and vascular area in the *PyMT;Col15a1^−/−^* mammary tumours, we postulated that, relative to controls, the knockout tumours have fewer vessels but are larger in size. This result is in accordance with the observations here and in other studies showing that ColXV expression is strong and persistent in the vasculature of human tumours [13,14,16] and is anticipated to play an important role in supporting the tumour vasculature. Ultrastructural analysis revealed locally thicker BMs and protein aggregates around capillaries in the *PyMT;Col15a1^−/−^* tumours, similar to what has been observed in heart capillaries [10]. Together, these observations further support the conclusion that this collagen plays an important role in vessel formation and in maintaining capillary integrity, both during organ development and in solid tumours. Our data from the *PyMT* mammary carcinoma and the MCA fibrosarcoma models also support the previous data that ColXV-derived restin is not an efficient inhibitor of tumour angiogenesis [25]. 

In conclusion, our data from the in vivo mammary carcinoma model support the previous evidence for the tumour suppressor role of ColXV and advocate that the effect of ColXV in tumourigenesis is not due to the anti-angiogenic properties of its restin domain but rather because of its protective effect on tumour ECM remodelling and subsequent consequences in tumour cell behaviour. The discrepancy in tumour growth that we observed between the genetic *MMTV-PyMT* model and the transplantable or chemical models may reflect the differences in ColXV function in cancers of distinct cell origin and regulation, as well as differences in the ability of different experimental models to accurately simulate functions of specific proteins. 

## 4. Materials and Methods

### 4.1. Mice and Licences

*Col15a1* knockout mice (*Col15a1^tm1Pih^*, MGI:2386162) have been generated previously [11]. These mice were backcrossed for at least eight generations into the FVB/N (Harlan, Horst, The Netherlands) background and for at least 10 generations into the C57BL/6J OlaHsd (Harlan). The knockout mice in the FVB/N background were further intercrossed with the *MMTV-PyMT* [FVB/N-Tg(MMTV-PyMT)634Mul/J] (MGI:2161653) mouse line (Jackson Laboratory, Bar Harbor, ME, USA) [41] for mammary carcinoma studies. 

Animal experiments were conducted in the Laboratory Animal Centre of the University of Oulu (OULAC) following the national and international legislation and guidelines for animal maintenance, care and experimentation. All mouse lines were maintained in a pathogen-free facility, group-housed in corncob bedding with enrichments, given ad libitum purified water and irradiated standard rodent chow and maintained on a 12:12-h light:dark cycle. *Col15a1* mouse lines were maintained by internal licences from the OULAC. Permissions and study protocols of tumour models were approved by the Oulu Provincial Government (syngeneic models, OLH-2002-790/Ym-23) and by the Finnish National Animal Experiment Board (licence numbers: ESLH-2007-09159/Ym-23, ESAVI/7328/04.10.03/2012 and ESAVI/1188/04.10.07/2016).

### 4.2. C57BL/6 Mouse Tumour Cell Lines

The B16F10 melanoma cell line was purchased from the American Type Culture Collection (ATCC Cat. No. CRL-6475), Lewis lung carcinoma cell line LL/2 (LLC1) was a generous gift from Prof. Karl Tryggvason, University of Oulu (origin ATCC Cat. No. CRL-1642) and T241 fibrosarcoma cell line [42] from Prof. Kari Alitalo, University of Helsinki. Cells were cultured in Dulbecco’s modified Eagle’s medium (DMEM), high glucose (4.5 g/L), supplemented with 2 mmol L-glutamine, penicillin (100 U/mL), streptomycin (100 mg/mL) and 10% foetal bovine serum (FBS). Cells were removed from culture plates with trypsin-EDTA (0.25%), harvested into DMEM containing 10% FBS to inactivate trypsin, washed twice with ice-cold phosphate buffered saline (PBS, pH 7.4) and re-suspended to serum-free DMEM to get a single-cell suspension of desired dilution for tumour transplantation studies. Cell viability was confirmed with Trypan blue exclusion and was typically approximately 90% for all cell lines.

### 4.3. MMTV-PyMT Mammary Carcinogenesis Model

*MMTV-PyMT* mouse lines were maintained by breeding the *PyMT* transgene-positive male mice, either wild type (*PyMT*) or ColXV knockout (*PyMT;Col15a1^−/−^*) with *PyMT*-negative wild-type females or *PyMT*-negative *Col15a1^−/−^* females, all in FVB/N background, to obtain female mice hemizygous for the *PyMT* transgene for mammary carcinogenesis experiments. Tumour formation in abdominal and thoracic mammary glands was monitored weekly with palpation to detect desmoplastic tissue starting at the age of 4 weeks and continuing until the age of 8, 10, 12 or 13 weeks in four *PyMT* and four *PyMT;Col15a1^−/−^* experimental groups, 5–10 females per group. If the size of a single mammary tumour or total tumour burden in an individual reached the pre-determined humane end-point criteria, the mouse was removed from the experiment. At the end of the monitoring period, mice were sacrificed with CO_2_ inhalation and cervical dislocation, and the four thoracic and two abdominal mammary glands were dissected, weighted, fixed with 4% paraformaldehyde (PFA) in PBS and embedded in paraffin or embedded in cryo-compound (Tissue-Tek OCT; Sakura, Alphen aan den Rijn, The Netherlands); and stored at −80 °C. 

### 4.4. MCA-Induced Fibrosarcoma

For fibrosarcoma induction, 9–11 weeks old *Col15a1^+/+^* and *Col15a1^−/−^* male mice in the C57BL/6J background were injected subcutaneously into both flanks with a freshly prepared solution of 3-methylchlolantrene (MCA; Merck, Darmstadt, Germany, Cat. No. 213942) in olive oil (100 mg/100 mL per mouse) under short isoflurane inhalation anaesthesia, as described earlier [43]. Tumour formation was monitored weekly for up to 20 weeks, first with palpations and later with gauge measurements. Subcutaneous tumours larger than 3 mm in diameter and showing progressive growth were counted as positive. Tumour volumes were calculated using the following formula: length × width^2^ × 0.52. At the end of the monitoring period, mice were sacrificed by CO_2_ inhalation and cervical dislocation, and tumours were dissected, fixed with 4% PFA and embedded in paraffin for tissue analyses.

### 4.5. Syngeneic Models

T241 fibrosarcoma cells (7 × 10^5^ per mouse), LLC1 lung carcinoma cells (7 × 10^5^ per mouse) and B16F10 melanoma cells (5 × 10^5^ per mouse) were injected subcutaneously into one flank of *Col15a1^−/−^* and *Col15a1^+/+^* male mice, all originating from breeding heterozygous *Col15a1^+/-^* mice in the C57BL6/J background. Rapid inhalation anaesthesia with isoflurane was employed to calm the mouse during the injection. Tumour growth was monitored at 2-day intervals with gauge measurements over the course of 3 weeks. At the end of the monitoring period, mice were sacrificed with CO_2_ inhalation and cervical dislocation, tumours were dissected, fixed with 4% PFA in PBS and embedded in paraffin.

### 4.6. Tissue Analyses

#### 4.6.1. Carmine Alum Staining on Whole Mounts

Tumour nodules in mammary ducts were assessed by fixing mammary gland whole mount preparations for 2 h in 4% PFA and staining them with 0.2% Carmine (Sigma-Aldrich, Darmstadt, Germany) and 0.5% aluminium potassium sulphate dodecahydrate in PBS (Sigma–Aldrich) overnight at room temperature (RT), as previously described [44]. The samples were then dehydrated through consecutive washes of increasing concentrations of ethanol, stored in methyl salicylate and imaged with a Leica DM LB2 light microscope equipped with a digital camera system (Leica, Wetzlar, Germany). For this analysis, 3–10 mammary glands per genotype were collected from mice at the age of 6, 9, 11 and 13 weeks. 

#### 4.6.2. Haematoxylin-Eosin Staining

The tissue morphology of mammary gland tumours was assessed by cutting PFA-fixed paraffin embedded tumour samples to 5 µm-thick sections, which were then stained with H&E and imaged with a light microscope (Leica DM LB2) equipped with a digital camera (Leica DFC 320). For this analysis, at least 10 mammary glands per genotype were collected from mice at the age of 6, 9, 11 and 13 weeks. 

#### 4.6.3. CD-31 Immunohistochemistry 

Blood vessels in PFA-fixed specimens were imaged with light microscopy using a rat monoclonal CD-31 antibody (Cat. No. 558736, BD Pharmingen, Franklin Lakes, NJ, USA) at 1:800 dilution in conjunction with a Tyramide Signal Amplification Biotin system (TSA Biotin Kit, Perkin Elmer Life Science, Waltham, MA, USA, Cat. No. NEL700001KT), following the detailed staining protocol provided by the manufacturer. Antigen retrieval was performed by boiling the samples for 15 min in 10 mM sodium citrate buffer (pH 6.0). Biotinylated secondary anti-rat antibody (Vector Laboratories, Oxfordshire, UK) was used at 1:250 dilution, streptavidin-conjugated horseradish peroxidase at 1:100 dilution and TSA reagent at 1:50 dilution. Stained sections were counterstained with Cole’s haematoxylin and mounted in Immumount (Thermo Scientific, Waltham, MA, USA). A Leica DM LB2 microscope with a digital camera (Leica DFC 320) was used for imaging.

#### 4.6.4. Analysis of Fibrillar Collagen Content

Masson’s trichrome and picrosirius red staining was performed to assess the content of fibrillar collagen in the mammary gland tumours. The PFA-fixed, 5 µm-thick tumour sections were dewaxed in xylene and rehydrated with decreasing ethanol series. According to the Masson’s basic protocol, the sections were treated with a nuclear stain (Celestine blue and Harris’ haematoxylin), followed by treatments with acid fuchsin and phosphomolybdic acid and, finally, with methyl blue stain (all reagents from Sigma–Aldrich). For picrosirius red staining, the sections were treated with 0.2% phosphomolybdic acid for 5 min followed by staining with 0.1% Direct Red 80/Sirius Red F3B (Sigma–Aldrich) in saturated picric acid for 1 h at RT. Both protocols were finalised by dehydration with ethanol series, clearing with xylene and mounting with Pertex (Sigma–Aldrich). The Masson’s trichrome-stained sections were imaged with a Leica DM LB2 microscope with a digital camera (Leica DFC320), and picrosirius red-stained sections were imaged under bright and polarised light with an Olympus BX51 microscope (Olympys, Tokyo, Japan) and a digital camera (Olympus DP71). Fiji ImageJ software was used for birefringence signal quantification and the ratio of thick versus thin collagen fibres was calculated, as described by Rasi et al. [10].

#### 4.6.5. Immunofluorescence Staining

For immunofluorescence staining, 5 µm-thick cryosections of mammary tumours were fixed for 20 min in methanol at −20 °C, air dried, washed twice for 5 min in PBS and then blocked with 1% BSA–0.3 M glycine in PBS–0.1% Tween 20 (PBST) for 1 h at RT in a humidified chamber. Samples were incubated with primary antibodies (Ab) diluted in 1% BSA–0.3 M glycine-PBST overnight at +4 °C, washed three times for 5 min in PBS and then incubated with fluorochrome-conjugated secondary antibodies and 4′6′-diamino-2-phenylindole (DAPI; Sigma-Aldrich) at 1:300 dilution for 1 h at RT in the dark. After three 5-min washes in PBS, the samples were mounted in Immu-Mount medium (Thermo Scientific). The following primary antibodies and secondary antibody combinations were used for immunofluorescence staining: polyclonal in-house rabbit ColXV Ab targeting the N-terminal non-collagenous portion of rabbit anti-mouse ColXV (83AA) [45] at 1:70 dilution, rabbit monoclonal Ki67 Ab (Cat. No. ab15590; Abcam, Cambridge, UK) at a concentration of 0.5 mg/mL with goat anti-rabbit Alexa Fluor 488 (Cat. No. A11008; Invitrogen, Waltham, MA, USA) Ab at 1:200 dilution, monoclonal rat anti-mouse CD-31 Ab (Cat. No. 550274; BD Biosciences, Franklin Lakes, NJ, USA) at 1:100 dilution with goat anti-rat Alexa Fluor 647 Ab (Jackson ImmunoResearch, West Grove, PA, USA) at 1:200 dilution, monoclonal mouse anti-rat PCNA (PC19, sc-56; Santa Cruz, Dallas, Tx, USA) at 1:100 dilution and Cy3-conjugated alpha smooth muscle actin (αSMA) Ab (Cat. No. C6198; Sigma Aldrich) at 1:200 dilution. A Zeiss LSM 780 confocal microscope (Zeiss, Jena, Germany) was used for immunofluorescence imaging.

#### 4.6.6. Morphometric Analyses

In mammary carcinoma samples, intratumoural CD-31 positive blood vessels were counted in tumours collected from nine 12-week-old females per genotype. Four microscopic fields per sample, representing the most vascularised areas of tumours, were counted at 100× magnification. Each branching structure was counted as a single vessel. ImageJ was used to determine the CD-31 positive areas in the same samples and microscopic fields. In the MCA model, 12 control tumours and 13 knockout tumours collected 13–17 weeks after tumour initiation with carcinogen treatment were included in the study. Ten microscopic fields at 200× magnification were counted per sample. The average number of Ki67- or PCNA-positive cells per sample was determined by ImageJ analysis software from 3 to 5 mice per genotype in each age group, and from 3 to 10 microscopic images per sample, depending on the size of the tumour and of areas containing proliferating cells. Results were calculated as percentages of Ki67- or PCNA-positive nuclei of the total number of nuclei in the image. In Supplemental Figure S1A,C, tumour samples from three to five mice per genotype and age were analysed (except there are no data on control *PyMT* mice at the age group 6–7 weeks due to bad quality of available cryosamples). 

The thickness of collagen fibres (thin, green; thick, red) was investigated from picrosirius red-stained samples via polarised light microscopy (Olympus). Original images were separated to RGB for automated thresholding using ImageJ image analysis software. Area fractions representing green and red colours were used to calculate ratios between the differentially stained collagen fibres. Collagen fibril diameter and density were manually assessed on 6800 x magnification TEM images using the Visiopharm^®^ software.

For assessing fibril density, 52–55 bundle regions/genotype, 5–14 bundle regions/tumour sample and 193–838 fibrils/tumour sample were measured and, for calculating fibril diameter fractions, 893–1032 fibrils/genotype and 121–265 fibrils/tumour sample were measured. Widths of protein aggregates on *PyMT;Col15a1^−/−^* tumour capillaries were measured at approximately 2 μm intervals on sites containing protein aggregation and lacking a clear BM structure on 4800x and 6800x magnification TEM images using Image J. Measurements were done in two *PyMT;Col15a1^−/−^* mice (two tumours samples/mouse), analysing 4–6 capillaries/tumor sample and performing 2–10 measurements/capillaries (resulting in total number of measurements, *n* = 104).

#### 4.6.7. Transmission Electron Microscopy

Approximately 1–2 mm^3^ samples of mammary glands from three 10-week-old *PyMT* and three *PyMT;Col15a1^−/−^* mice were fixed with 1% glutaraldehyde and 4% formaldehyde in 0.1 M phosphate buffer (pH 7.4), post-fixed in 1% osmium tetroxide, dehydrated in acetone and embedded in Epon LX 112 (Ladd Research Industries, Williston, VT, USA). Approximately 80 nm sections were examined using a Tecnai G2 Spirit transmission electron microscope, and images were captured using Veleta or Quemesa CCD cameras (Olympus Soft Imaging Solutions).

### 4.7. Kaplan-Meier Survival Analysis

Kaplan–Meier survival analysis was performed by accessing *COL15A1* gene expression data (Affymetrix *COL15A1* probe set 203477_at) of approximately 5000 breast cancer patients utilising the Kaplan–Meier plotter tool on the website www.kmplot.com (accessed on 14 september 2021) [31].

### 4.8. Statistical Analyses

Statistical significance of the differences between the groups was evaluated by a two-tailed Student’s *t*-test. *p* values of <0.05 were considered statistically significant (symbols in figures: *, *p* < 0.05; **, *p* < 0.01; ***, *p* < 0.001). Results are expressed as means ± the standard error of the means (S.E.M). Statistical analyses were done with the GraphPad Prism software.

## Supplementary Materials

The following are available online at https://www.mdpi.com/article/10.3390/ijms22189978/s1.
