# Supplementary material for: Deletion of Col15a1 Modulates the Tumour Extracellular Matrix and Leads to Increased Tumour Growth in the MMTV-PyMT Mouse Mammary Carcinoma Model
Source: Int J Mol Sci. 2021 Sep 15;22(18):9978. doi: 10.3390/ijms22189978 (PMC8467152; doi:10.3390/ijms22189978)
Supplement: Supplementary file 1 [file ijms-22-09978-s001.zip › ijms-1292048-supplementary.pdf]

Supplementary Material

Deletion of *Col15a1* Modulates the Tumour Extracellular Matrix and Leads to Increased Tumour Growth in the *MMTV-PyMT* Mouse Mammary Carcinoma Model

Guillermo Martínez-Nieto, Ritva Heljasvaara, Anne Heikkinen, Hanne-Kaisa Kaski, Raman Devarajan, Otto Rinne, Charlotta Henriksson, Emmi Thomson, Camilla von Hertzen, Ilkka Miinalainen, Heli Ruotsalainen, Taina Pihlajaniemi and Sanna-Maria Karppinen

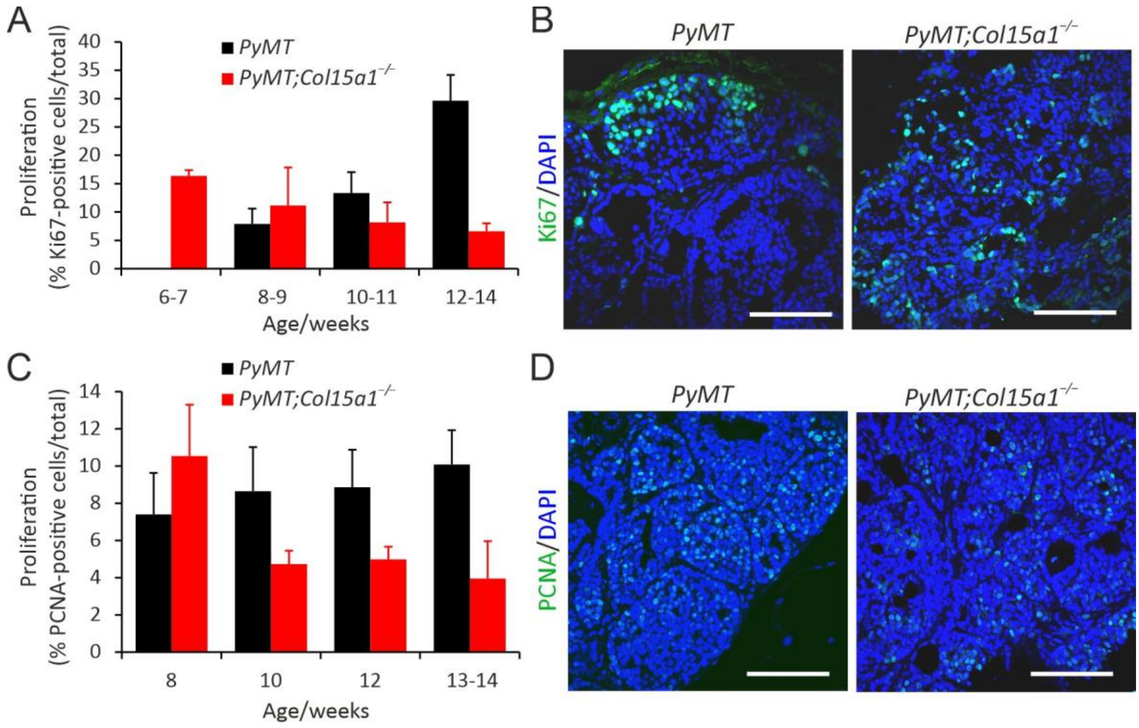

Supplementary Figure S1. Analysis of proliferative cells in *PyMT;Col15a1*$^{-/-}$ and control *PyMT* mouse mammary tumours. (A) Quantification of Ki67-positive cells and (B) representative images of Ki67 immunofluorescence staining. (C) Quantification of PCNA-positive cells and (D) representative PCNA immunofluorescence staining. The mean percentages of positive nuclei of the total number of nuclei are presented. Three to seven microscopic fields per section were analysed. Immunofluorescence stainings are from mammary tumours at week 12. Scale bar, 200 μm. Fluorescent signals: Ki67 or PCNA, green; nuclei, DAPI, blue.

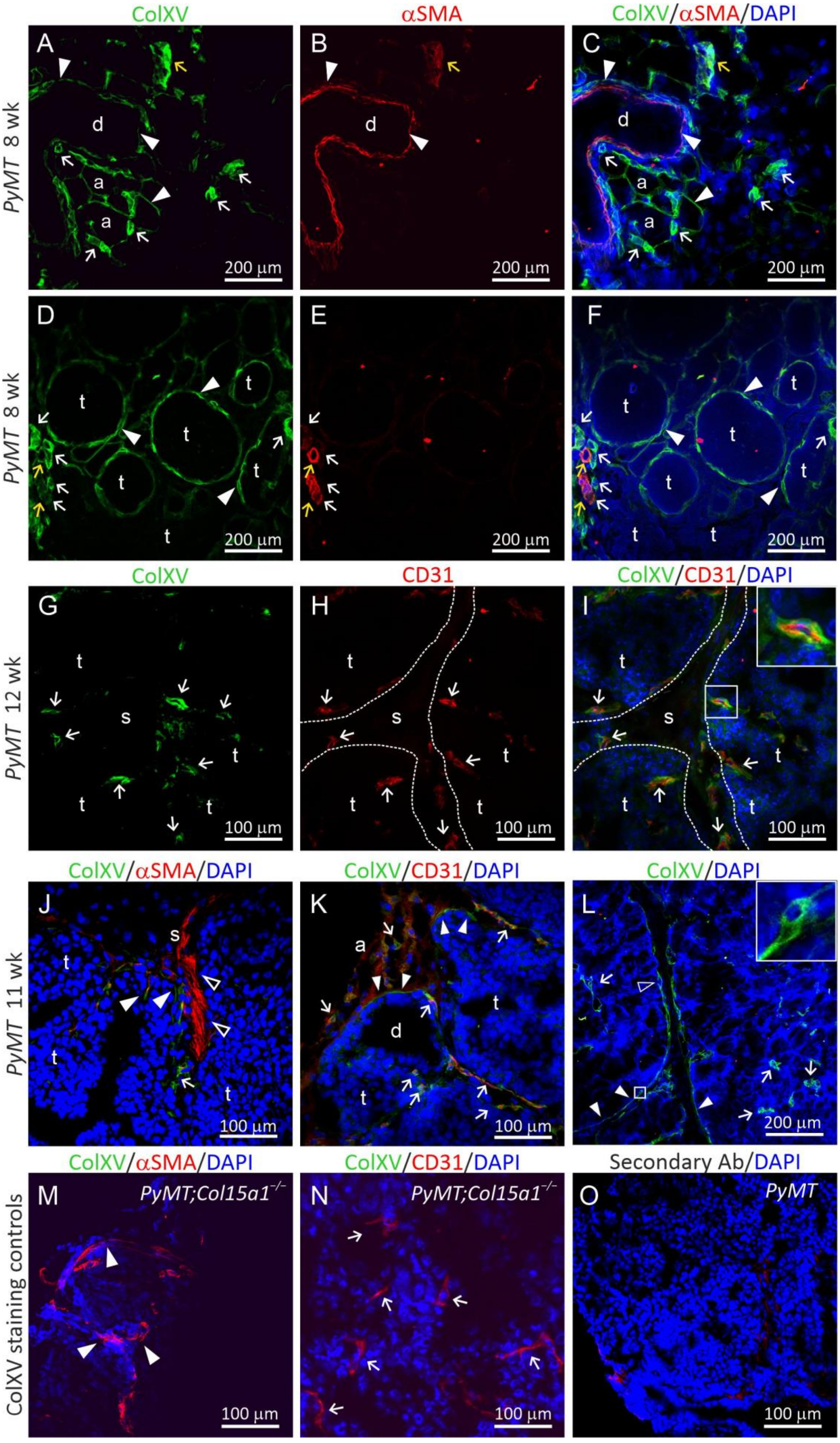

ColXV
αSMA
ColXV/αSMA/DAPI
PyMT 8 wk
PyMT 8 wk
ColXV
CD31
ColXV/CD31/DAPI
PyMT 12 wk
ColXV/αSMA/DAPI
ColXV/CD31/DAPI
ColXV/DAPI
PyMT 11 wk
ColXV/αSMA/DAPI
ColXV/CD31/DAPI
Secondary Ab/DAPI
ColXV staining controls
PyMT;Col15a1−/−
PyMT;Col15a1−/−
PyMT
A
B
C
D
E
F
G
H
I
J
K
L
M
N
O
d
a
t
s
200 μm
100 μm

Supplementary Figure S2. Immunofluorescence microscopy of ColXV expression and localisation in mouse mammary tumours. (A–F) Double staining for ColXV and alpha smooth muscle actin (αSMA) in *PyMT* tumours. (A–C) ColXV (green) localised to normal ductal (d) basement membrane (BM) (arrowhead), adjacent to tumour tissue, to BMs of blood vessels and capillaries (arrow), and to the thin BM around adipocytes (a). αSMA (red) localised to the myoepithelial cell layer in healthy ducts and to the smooth muscle layers of vessels (yellow arrow). (D–F) In early-stage tumours, ColXV localises around tumour (t) nests (arrowhead) and to vascular BMs (arrow). In larger vessels, the ColXV signal co-localises with αSMA (yellow arrow). (G–I) Double staining for ColXV and CD-31 in an invasive mammary tumour. The ColXV signal associates with vessels, in close proximity with the endothelial cell marker CD-31 (arrow). ColXV is mainly absent in the borders of the invasive tumours, marked with a dashed line in H and I. The insert in I shows that ColXV is localised to the BM zone surrounding the endothelium in vessels. (J) On some occasions, a faint and fragmented ColXV signal surrounds the tumour nest in invasive mammary tumours and associates with the αSMA signal (white arrowhead). ColXV is also present in tumour vessels (arrow). In the desmoplastic tumour stroma (s), strong αSMA staining in elongated cancer-associated fibroblasts is evident (open arrowhead). (K) Another example of a mammary tumour in which ColXV surrounds a partially filled mammary duct (arrowhead) and co-localises with CD-31 in blood vessels. (L) In addition to the thin BM structures lining the tumour nests (white arrowhead) and the vascular BM (arrow), ColXV shows cytoplasmic staining in myoepithelial cells surrounding the tumour islands (open arrowhead and insert in L) in the early phase of tumourigenesis. (M-O) Controls. Double immunofluorescence staining for (M) ColXV (green) and αSMA (red), and (N) ColXV (green) and CD31 (red) in ColXV-deficient *PyMT;Col15a1*$^{-/-}$ mammary tumours. αSMA signal in the myoepithelial layer (arrowheads) and CD31 signal in tumour vasculature (arrow) are evident and ColXV staining is negative, showing the specificity of the anti-ColXV antibody [45] used in the study. (O) Control (without primary antibody) for ColXV/αSMA staining indicating nonspecific binding of the secondary antibody. a, adipocyte; d, mammary duct; s, tumour stroma; t, tumour tissue. Fluorescent signals: ColXV, green; αSMA or CD-31, red; nuclei, DAPI, blue
